# Detection-dependent six-photon Holland-Burnett state interference

**DOI:** 10.1038/srep36914

**Published:** 2016-11-14

**Authors:** Rui-Bo Jin, Mikio Fujiwara, Ryosuke Shimizu, Robert J. Collins, Gerald S. Buller, Taro Yamashita, Shigehito Miki, Hirotaka Terai, Masahiro Takeoka, Masahide Sasaki

**Affiliations:** 1 National Institute of Information and Communications Technology (NICT), 4-2-1 Nukui-Kitamachi, Koganei, Tokyo 184-8795, Japan; 2 School of Science and Laboratory of Optical Information Technology, Wuhan Institute of Technology, Wuhan 430205, China; 3 University of Electro-Communications (UEC), 1-5-1 Chofugaoka, Chofu, Tokyo 182-8585, Japan; 4 SUPA, Institute of Photonics and Quantum Sciences, School of Engineering and Physical Sciences, Heriot-Watt University, Edinburgh EH14 4AS, United Kingdom; 5 National Institute of Information and Communications Technology (NICT), 588-2 Iwaoka, Kobe 651-2492, Japan

## Abstract

The NOON state, and its experimental approximation the Holland-Burnett state, have important applications in phase sensing measurement with enhanced sensitivity. However, most of the previous Holland-Burnett state interference (HBSI) experiments only investigated the area of the interference pattern in the region immediately around zero optical path length difference, while the full HBSI pattern over a wide range of optical path length differences has not yet been well explored. In this work, we experimentally and theoretically demonstrate up to six-photon HBSI and study the properties of the interference patterns over a wide range of optical path length differences. It was found that the shape, the coherence time and the visibility of the interference patterns were strongly dependent on the detection schemes. This work paves the way for applications which are based on the envelope of the HBSI pattern, such as quantum spectroscopy and quantum metrology.

Multi-photon entanglement and multi-photon interference are useful nonclassical phenomena in quantum information applications^1^,^2^. In particular, the so-called NOON state interference (NOON-SI) is a powerful tool to improve the precision of phase sensing measurement. The NOON state is a path-entangled state with *N* photons occupying either one of two optical paths^3^: 
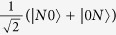
. Ideal NOON states are challenging to prepare in experiments and therefore Holland-Burnett (HB) states are traditionally used to approximate the NOON state^4^,^5^. The HB state can be easily prepared in experiments using photon pairs from spontaneous parametric down conversion (SPDC). By passing a 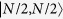 initial state through a beamsplitter and introducing a phase shift of *ϕ*, the HB state can be generated^5^ in the form of 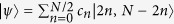, with 
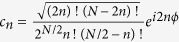
. HB states approximate the NOON state^6^ in that they can attain the Heisenberg limit for phase estimation, but the photon number exhibits a quadratic variance in *N*. It has been shown that HB states are almost optimally robust to imperfect state preparation and detection, and losses^7^.

The NOON state, and its experimental approximation of the HB state can be used to perform measurements with enhanced sensitivity and, therefore, have been widely used in quantum lithography^3^, quantum metrology^8^, quantum microscopy^9^,^10^, and error correction^11^. Many HB state interference (HBSI) experiments have been carried out with photon numbers (values of *N*) from two^12^ to four^13^,^14^, and six^5^ both at visible and optical fiber telecommunications wavelengths^15^,^16^.

All these previous HBSI schemes mainly considered the period-based applications and only measured the single-mode interference pattern, which comprises solely of the portion of the interference patterns around zero optical path length difference. As a result, an analysis of the full-range properties of the HBSI, e.g., the overall envelope shape and the coherence time, was omitted. Therefore, although detailed in their analysis of the single-mode interference patterns, the previous experiments are not enough to fully characterize the HBSI, especially for higher photon numbers (*N* > 2). In this work, we consider the properties of the HBSI pattern over a wide range of optical path length differences and experimentally measured HBSI patterns up to six photons, generated at a wavelength in the optical fiber telecommunications L-band by a SPDC source. It was found that the shape, the coherence time and the visibility of the interference patterns were strongly dependent on the detection schemes. Inspired by the theoretical work by Tichy *et al*. for detection dependent Hong-Ou-Mandel-type multiphoton interference^17^,^18^,^19^,^20^,^21^, we further developed this theory for HB-state-type multiphoton interference in this work. The theoretical simulations correspond well with the experimental results. Further, the effects of multi-photon emission in HBSI are analyzed in detail. The experiment and theory described in this work are useful for the future envelope-based applications, such as quantum spectroscopy^22^,^23^ and quantum metrology^24^,^25^.

## Experiment and Results

**Figure 1:**
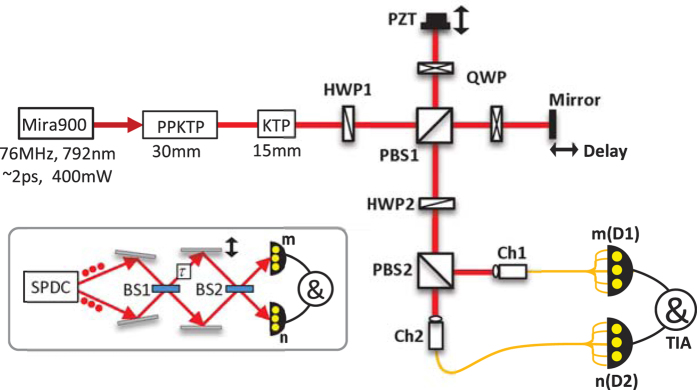
The experimental setup. HWP = half wave plate, QWP = quarter wave plate, PZT = piezo-electric linear actuator, PBS = polarization beam splitter, TIA = time interval analyzer. The inset depicts a standard configuration of the HB state interference using path-mode.

Our experimental setup for multi-photon NOON-SI is shown in Fig. 1. A summary of the experiment will be presented here while a more detailed description of the components will be presented in the Methods. 792 nm wavelength laser pulses of temporal duration 2 ps emitted from a mode-locked Titanium sapphire laser (Mira900, Coherent Co.) at a pulse repetition frequency of 76 MHz pumped a 30-mm-long, 46.1 *μ*m poling period potassium titanyl phosphate (KTP) crystal for type-II group-velocity-matched SPDC^26^,^27^. The periodically poled KTP (PPKTP) crystal was temperature controlled to 32.5 ± 0.1 °C to maintain a stable output. The down converted photons, i.e., the signal and idler, had orthogonal polarizations and degenerate wavelengths at 1584 nm. To compensate for their different group velocities during propagation through the nonlinear crystal, the downconverted biphotons passed through a KTP crystal of half the length of the previous crystal (15 mm), which was maintained at room temperature (20 °C). After the biphotons were mixed on a half wave plate (HWP1, at 22.5°), they were sent to a time scanning system, which was composed of a polarization beam splitter (PBS1), two quarter wave plates (QWP, at 45°) and two mirrors. One of the mirrors was mounted on a piezo-electric (PZT) linear actuator to achieve a scanning step in the order of nm, while the other one was mounted on a stepping motor linear actuator to realize a scanning step of the order of *μ*m. Then, the biphotons were mixed again on the second half wave plate (HWP2, at 22.5°) and separated by PBS2, before they were collected into two channels composed of single-mode fibers (Ch1 and Ch2). Ch1 was connected to a 1 × *m* fiber coupler and each of the *m* output ports was coupled to a superconducting nanowire single-photon detector (SNSPD)^28^,^29^,^30^, which had a dark count rate of less than 1 kilo counts per second (kcps). Ch2 was treated similarly but here a 1 × *n* fiber coupler and *n* SNSPDs are considered. Finally, all the detected events from every SNSPD were sent to a time interval analyzer (TIA) for coincidence counting.

**Figure 2:**
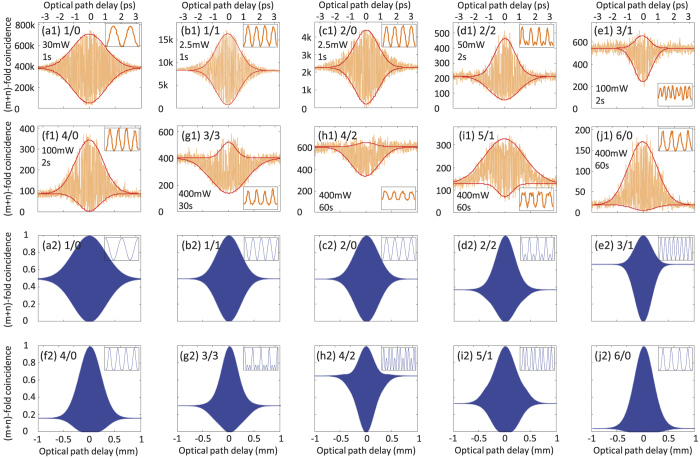
Experimental results and numerical simulations of multi-photon HBSI under the *m*/*n* detection schemes. The first two rows (a1–j1) are the experimental results obtained by scanning a stepping motor for 1000 steps with a step length of 2 *μ*m. The parameters on the left side are the pump power (in mW) and accumulation time (in seconds, for each point). The inset in each figure is the interference pattern obtained by scanning a Piezo (PZT) near the zero delay position. The horizontal axis for each inset is phase delay from 0 to 4*π*, while the vertical axis is the same as each main figure. The third and fourth rows (a2–j2) are the corresponding numerical simulations using the theoretical model. Insets in (a2–j2) are the fine interference patterns near the zero delay position.

By changing the different fiber couplers, different *m*/*n* detection schemes could be examined. A total of six SNSPDs were employed in the experiments reported here and the 1/0, 1/1, 2/0, 2/2, 3/1, 4/0, 3/3, 4/2, 5/1 and 6/0 detection results are shown in Fig. 2(a1–j1).

All the data in Fig. 2(a1–j1) are presented as raw data without subtracting any background counts. It is clearly shown in Fig. 2 that the profile, the coherence time and the visibility of the interference patterns are dependent on the detection schemes. Figure 2(a1) shows the one-photon HBSI, with only the signal photons input and only one SNSPD for detection. The idler photons are blocked by inserting a PBS after the PPKTP crystal. To overcome the problem of dark counts in the SNSPD, we utilized a pump power of 30 mW for the one-photon interference. Figure 2(b1–c1) shows the two-photon HBSI with the detection schemes of 1/1 and 2/0. Figure 2(d1–f1) are the four-photon HBSI patterns with the detection schemes of 2/2, 3/1 and 4/0. Figure 2(g1–j1) are the six-photon HBSI patterns with the detection schemes of 3/3, 4/2, 5/1 and 6/0. To obtain the experimental data in a reasonable time, we adopted a high pump power of 400 mW for the six-photon interference. The mean photon numbers at different pump powers were about 0.04 (30 mW), 0.067 (50 mW), 0.15 (100 mW) and 0.64 (400 mW) respectively and the multi-pair components in our PPKTP source as a function of pump power were investigated in our previous work^31^.

We can quantitatively evaluate the interference patterns in Fig. 2(a1–j1) by using three different parameters: the shape, the coherence length and the visibility, as shown in Table 1. We classify the shape of the interference patterns into three categories: symmetric profile, dip or bump. The 1/0, 1/1 and 2/0 detection schemes have a symmetric profiles, since the upper envelope and the lower envelope have the same bandwidth. The 2/2, 4/0, 5/1 and 6/0 detection schemes show bump profiles while the profiles for the 3/1, 3/3 and 4/2 detection schemes are dips. The coherence length (bandwidth) of bumps are defined as the full-width at half maximum (FWHM) of the upper envelope, while the bandwidth of the dips are defined as the FWHM of the lower envelopes. The 3/1 detection scheme has the smallest bandwidth, while 5/1 has the largest bandwidth. The coherence time is directly calculated from coherence length by dividing the speed of light. The visibilities are calculated from the nm scale scanning step data in the inset in Fig. 2(a1–j1). The visibility of 4/2 schemes is the lowest visibility, while the 1/0, 2/0, 4/0 and 6/0 schemes always maintain high visibilities, even at high pump powers.

### Theoretical analysis

To fully explain the experimental results, we developed a multi-mode theory using Schmidt decomposition on the temporal modes of the HB state. Our theory is inspired by the previous multi-mode theory by Tichy *et al*. for Hong-Ou-Mandel type interference^17^,^18^,^19^,^20^,^21^. The calculated detection probability *P*_*mn*_ for each *m*/*n* detection schemes is:


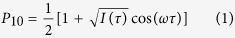



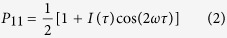



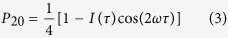



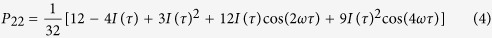



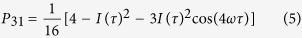



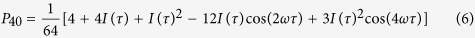


where, *τ* is the optical path delay, *ω* is the angular frequency, and


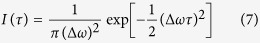


is the indistinguishability^19^, with Δ*ω* corresponding to the spectral width of the photon source. See the Supplementary Information for more details regarding the derivation of these equations, and the *P*_*mn*_ for six-photon detection schemes. It should be emphasized that the HBSI is different from the Hong-Ou-Mandel (HOM) type interference, for example, the HBSI scheme has two beamsplitters and is phase sensitive, while the HOM type interference only has one beamsplitter and is phase insensitive. So, the theoretical calculation of the HBSI is different from the previous works for HOM type interferometry. To the best of our knowledge, the equations presented above represent the first analysis of multi-mode HBSI.

From the experimentally measured bandwidth in the 1/1 and 2/0 schemes in Table 1, we can estimate the Δ*ω* value in Eq. (7). With the Δ*ω* value and the equations of *P*_*mn*_, we plot the theoretically expected interference patterns in Fig. 2(a2–j2). While the two-photon schemes (1/1 and 2/0 detection) have the same profiles, i.e. the profiles are detection-independent, for the four-photon and six-photon schemes the theoretical patterns are completely detection-dependent, i.e. different *m*/*n* detection schemes have different profiles. The visibilities for all the simulated patterns in Fig. 2(a2–j2) are normalized. The main figures and insets in Fig. 2(a2–f2) correspond well with the experimental results in Fig. 2(a1–f1). However, for the six-photon cases in Fig. 2(g2–j2) and Fig. 2(g1–j1), there are several discrepancies for both the main figures and the insets. This was mainly caused by the strong multi-pair emission attributable a high pump power of 400 mW. The effect of multi-pair emission will be discussed in detail in the following section.

## Discussion

**Figure 3 f3:**
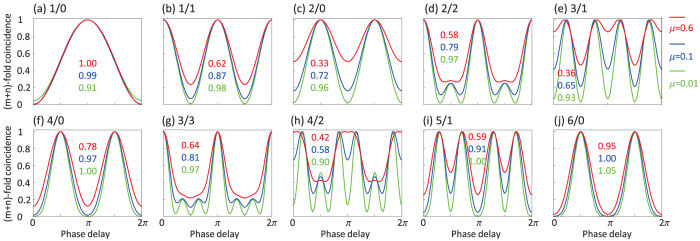
Analysis of multi-pair emission. Theoretical multi-photon HBSI patterns with mean photon numbers of 0.01 (green), 0.1 (blue) and 0.6 (red). The visibilities are also labeled in the figure.

The multi-pair emission is an important reason for the discrepancy between the theoretical simulations and the experimental results in Fig. 2. To obtain a reasonable counts for six photon detections in a short time, we applied a high pump power of 400 mW, which inevitably induced higher-order emissions. To analyze the effect of multi-pair emission in the interference patterns, we constructed a model of our experiment using the characteristic functions method, which was previously used in entanglement swapping analysis^32^,^33^. In this model, the SPDC source is considered as a squeezed vacuum and all the multi-pair components are included. Further, the collection efficiency *η*, dark counts *dc* and mean photon numbers *μ* are also included in our model. The coincidence probability *p*_*mn*_ can be written as


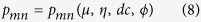


where *μ* is the mean photon number per pulse (also the squeezing parameter), *η* is the total efficiency (the product of collection efficiency and detector efficiency), *dc* is the dark count probability for one counting event, and *ϕ* is the phase delay. With *η* = 0.2, *dc* = 0.0001, we plot the *p*_*mn*_ as a function of the phase delay *ϕ* for *μ* value of 0.01, 0.1, 0.6, as shown in Fig. 3(a–j).

In Fig. 3, the 4/0 and 6/0 schemes maintain relatively high visibilities, even for high mean photon numbers. This phenomenon is also verified by the experimental results in Fig. 2. In contrast, the visibilities of the 3/1 and 4/2 schemes decrease dramatically for higher mean photon numbers. Further, the higher frequency fringes in the 3/1 and 3/3 schemes (which are 4 times and 6 times higher frequency of the 1/0 scheme, respectively) are very important for high-precision phase sensing^5^,^14^. However, these high frequency fringes degrade rapidly at high *μ* values. Therefore, it is very important to maintain a low *μ* values for phase sensing applications of NOON state. In Fig. 2, under the same pump power, the 2/0 scheme has a higher visibility than the 1/1 scheme. This result is different from our theoretical expectation in Fig. 3. This discrepancy may be caused by the spatial mode matching condition in the experiment. To solve this problem in the future, more parameters should be included in the theoretical model.[Fig f3]

It is necessary to compare the interference pattern of a HB state with that of a NOON state. A HB state shows detection dependency, however a NOON state does not show such a detection dependent phenomenon. For example, in the four-photon case, a HB state can be written as 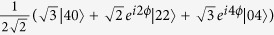, while a NOON state with phase shift can be written as 
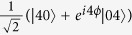
. For this NOON state, the coincidence probability in the 2/2, 3/1, and 4/0 detection schemes can be calculated as 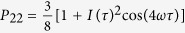, 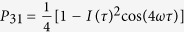 and 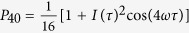. Clearly, the coherence time, the envelope shape and the oscillation frequency are the same for these three different detection schemes. This results are completely different from the ones for the HB state. It is the extra term of 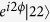 in the HB state that caused such a detection dependency.

In our experiment, the detection efficiencies of the six detectors are different, but the spread in detection efficiencies is not responsible for the shape of the envelopes reported in this work as they only affect the rate of coincidence counts recorded. It is possible to extend our scheme to 8-photon or higher HBSI, if more efficient sources and detectors are available. Especially, it is necessary to decrease the multi-photon emission, and one possible to way is to increase the repetition rate of the pump laser^32^, which can decrease the multi-pair components without decreasing the count rates.

In the future, this experiment may be used for applications which are based on the envelope of the HBSI pattern, for example, in quantum spectroscopy^22^,^23^ and quantum metrology^24^,^25^, such a time-domain full-range-characterized interference patterns may contain useful spectral information of a sample after transmission or reflection by the sample.

## Conclusion

We experimentally and theoretically demonstrated six-, four- two- and one-photon HBSI with a spontaneous parametric down conversion source and six superconducting nanowire single-photon detectors. It was found that the shape, the coherence time and the visibility of the interference patterns are strongly dependent on the detection schemes. This is the first experimental observation of detection dependency of HBSI up to 6 photons at telecom wavelengths. This experiment can be used for applications which are based on the envelope of the HBSI pattern, such as quantum spectroscopy and quantum metrology.

## Methods

### The SPDC

In this experiment, the SPDC source is based on a PPKTP crystal, which satisfies the group-velocity-matched condition at telecom wavelength^26^,^27^,^34^,^35^,^36^,^37^,^38^. Thanks to the GVM condition, the spectral purity is much higher at telecom wavelength than that at visible wavelengths^39^. The multi-pair components in our PPKTP source as a function of pump power were investigated in our previous work^31^.

### The SNSPDs

The superconducting nanowire single photon detectors (SNSPDs) in this experiment are fabricated with 5–9 nm thick and 80–100 nm wide niobium nitride (NbN) or niobium titanium nitride (NbTiN) meander nanowires on thermally oxidized silicon substrates^28^,^29^,^30^. The nanowire covers an area of 15 *μ*m × 15 *μ*m. Our SNSPDs are installed in a Gifford-McMahon cryocooler system and operate at a temperature of 2.1 Kelvin. We used six SNSPDs in this experiment. When biased such that the dark counts were less than 1 kcps, the detection efficiencies were about 70%, 70%, 60%, 60%, 46% and 29% for these six detectors.

## Additional Information

**How to cite this article**: Jin, R.-B. *et al*. Detection-dependent six-photon Holland-Burnett state interference. *Sci. Rep*. **6**, 36914; doi: 10.1038/srep36914 (2016).

**Publisher’s note**: Springer Nature remains neutral with regard to jurisdictional claims in published maps and institutional affiliations.

## Supplementary Material

Supplementary Information

## Figures and Tables

**Table 1:** Parameters of the experimental patterns in Fig. 2(a1–j1).

Detection scheme *m*/*n*	1/0	1/1	2/0	2/2	3/1	4/0	3/3	4/2	5/1	6/0
Profile shape	sym.	sym.	sym.	bump	dip	bump	dip	dip	bump	bump
Coherence length (mm)	0.75	0.53	0.53	0.46	0.40	0.63	0.81	0.62	0.92	0.65
Coherence time (ps)	2.5	1.77	1.77	1.53	1.33	2.10	2.70	2.07	3.07	2.17
Visibility	0.99	0.92	0.98	0.85	0.53	0.98	0.63	0.35	0.73	0.98

sym. = symmetric shape.
