# Supplementary material for: Detection-dependent six-photon Holland-Burnett state interference
Source: Sci Rep. 2016 Nov 14;6:36914. doi: 10.1038/srep36914 (PMC5378926; doi:10.1038/srep36914)
Supplement: Supplementary Information [file srep36914-s1.pdf]

Supplemental Information for
Detection-dependent six-photon Holland-Burnett state interference

Rui-Bo Jin, Mikio Fujiwara, Ryosuke Shimizu, Robert J. Collins, Gerald S. Buller, Taro Yamashita, Shigehito Miki, Hirotaka Terai, Masahiro Takeoka, and Masahide Sasaki

Multi-mode theory for Holland-Burnett state interference

Here we deduce the equations for the Holland-Burnett (HB) state interference using multi-mode theory. The key feature of this theory is the Schmidt decomposition on the temporal modes, which was first proposed by Tichy et al for a Hong-Ou-Mandel type multi-photon interference [1–3]. Here, we further develop the theory for the HB-state type multi-photon interferences. The setup of the HB state interference is shown in Fig. 1.

Two-photon HB state interference

First, we provide the derivation for the two-photon case. The input state is

$$|1,1\rangle_{s,i} = \hat{a}_s^\dagger \hat{b}_i^\dagger |0\rangle , \tag{1}$$

where $\hat{a}_s^\dagger$ and $\hat{b}_i^\dagger$ are the creation operator for the signal and idler photons from spontaneous parametric downconversion (SPDC). After the first beamsplitter (BS1), the state evolves to be

$$\frac{1}{2}[(\hat{a}_0^\dagger)^2 - (\hat{b}_0^\dagger)^2] |0\rangle \tag{2}$$

where, $\hat{a}_s^\dagger = (\hat{a}_0^\dagger + \hat{b}_0^\dagger)/\sqrt{2}$, and $\hat{a}_i^\dagger = (\hat{a}_0^\dagger + \hat{b}_0^\dagger)/\sqrt{2}$. After a phase shift $\omega\tau$ in the arm a_0, e.g. by scanning the optical path delay τ, the state evolves to be

$$\frac{1}{2}[(e^{i\omega\tau}\hat{a}_0^\dagger(t))^2 - \hat{b}_0^\dagger(t+\tau)^2] |0\rangle \tag{3}$$

In the above equation, due to the optical path delay between the arm a_0 and b_0, the time information for $\hat{a}_0^\dagger$ is t, different from the time information for $\hat{b}_0^\dagger$ of $t+\tau$. We can apply a Schmidt decomposition on the temporal modes

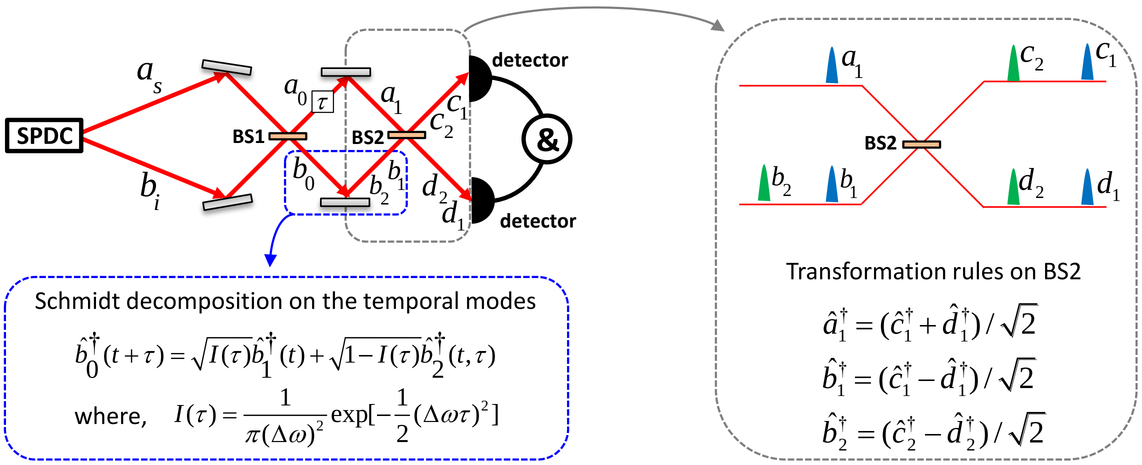

FIG. 1: The setup of HB state interference. τ = optical path delay. & = coincidence counter.

for arm b_0:

$$\hat{b}_0^\dagger(t+\tau) = \sqrt{I(\tau)}\hat{b}_1^\dagger(t) + \sqrt{1-I(\tau)}\hat{b}_2^\dagger(t,\tau), \tag{4}$$

and obtain the state of

$$\frac{1}{2}[e^{i2\omega\tau}\hat{a}_1^\dagger(t)^2 - (\sqrt{I(\tau)}\hat{b}_1^\dagger(t) + \sqrt{1-I(\tau)}\hat{b}_2^\dagger(t,\tau))^2]\,|0\rangle \tag{5}$$

where $\hat{a}_0^\dagger(t) = \hat{a}_1^\dagger(t)$ and

$$I(\tau) = \frac{1}{\pi(\Delta\omega)^2}\exp[-\frac{1}{2}(\Delta\omega\tau)^2] \tag{6}$$

is the indistinguishability. $\Delta\omega$ is determined by the spectral width of the photon source.

Then, the photons passed through the second beamsplitter (BS2), with transformation rule of

$$\hat{a}_1^\dagger = (\hat{c}_1^\dagger + \hat{d}_1^\dagger)/\sqrt{2}, \tag{7}$$
$$\hat{b}_1^\dagger = (\hat{c}_1^\dagger - \hat{d}_1^\dagger)/\sqrt{2}, \tag{8}$$
$$\hat{b}_2^\dagger = (\hat{c}_2^\dagger - \hat{d}_2^\dagger)/\sqrt{2}. \tag{9}$$

The resultant state is

$$\frac{1}{4}\{e^{i2\omega\tau}(\hat{c}_1^\dagger + \hat{d}_1^\dagger)^2 - [\sqrt{I}(\tau)(\hat{c}_1^\dagger - \hat{d}_1^\dagger) + \sqrt{1-I(\tau)}(\hat{c}_2^\dagger - \hat{d}_2^\dagger)]^2\}\,|0\rangle\,. \tag{10}$$

In the above equation, we omit the time label for each operator for simplicity, i.e. $\hat{c}_1^\dagger = \hat{c}_1^\dagger(t)$, $\hat{d}_1^\dagger = \hat{d}_1^\dagger(t)$, $\hat{c}_2^\dagger = \hat{c}_2^\dagger(t,\tau)$, and $\hat{d}_2^\dagger = \hat{d}_2^\dagger(t,\tau)$.

For $|1,1\rangle$ state detection at ports c and d: the corresponding projected state is

$$\frac{1}{2}\{e^{i2\omega\tau}\,|11\rangle_{c_1d_1} + I(\tau)\,|11\rangle_{c_1d_1} + [1-I(\tau)]\,|11\rangle_{c_2d_2} + \sqrt{I(\tau)[1-I(\tau)]}(|11\rangle_{c_1d_2} + |11\rangle_{c_2d_1})\}. \tag{11}$$

The detection probability of $|1,1\rangle$ state is

$$P_{11} = \frac{1}{4}\{|e^{i2\omega\tau} + I(\tau)|^2 + |1-I(\tau)|^2 + 2|\sqrt{I(\tau)[1-I(\tau)]}|^2\} = \frac{1}{2}[1 + I(\tau)\cos(2\omega\tau)] \tag{12}$$

For $|2,0\rangle$ state detection at port c: the corresponding projected state is

$$\frac{1}{4}\{\sqrt{2}e^{i2\omega\tau}\,|20\rangle_{c_1c_1} - \sqrt{2}I(\tau)\,|20\rangle_{c_1c_1} - \sqrt{2}[1-I(\tau)]\,|20\rangle_{c_2c_2} - 2\sqrt{I(\tau)[1-I(\tau)]}\,|20\rangle_{c_1c_2}\} \tag{13}$$

The detection probability of $|2,0\rangle$ state is

$$P_{20} = \tfrac{1}{8}\{|e^{i2\omega\tau} - I(\tau)|^2 + |1-I(\tau)|^2 + 2|\sqrt{I(\tau)[1-I(\tau)]}|^2\} = \tfrac{1}{4}[1 - I(\tau)\cos(2\omega\tau)] \tag{14}$$

2n-photon HB state interference

Following the same procedure, we can also calculate the detection probability for the cases of four-photon, six-photon and 2n-photon input state. Further, this method can be used to calculate the detection probability for one-photon Mach-Zehnder interference p_{10}. We summarize the detection probability for up to six-photon as follows:

$$P_{10} = \frac{1}{2}[1 + \sqrt{I}\cos(\omega\tau)] \tag{15}$$

$$P_{11} = \frac{1}{2}[1 + I\cos(2\omega\tau)] \tag{16}$$

$$P_{20} = \frac{1}{4}[1 - I\cos(2\omega\tau)] \tag{17}$$

$$P_{22} = \frac{1}{32}[12 - 4I + 3I^2 + 12I\cos(2\omega\tau) + 9I^2\cos(4\omega\tau)] \tag{18}$$

$$P_{31} = \frac{1}{16}[4 - I^2 - 3I^2\cos(4\omega\tau)] \tag{19}$$

$$P_{40} = \frac{1}{64}[4 + 4I + I^2 - 12I\cos(2\omega\tau) + 3I^2\cos(4\omega\tau)] \tag{20}$$

$$P_{33} = \frac{3}{512}[40 - 24I + 18I^2 + I(48 - 18I + 15I^2)\cos(2\omega\tau) + 30I^2\cos(4\omega\tau) + 25I^3\cos(6\omega\tau)] \tag{21}$$

$$P_{42} = \frac{3}{512}[40 - 8I - 6I^2 + I(16 + 8I - 15I^2)\cos(2\omega\tau) - 10I^2\cos(4\omega\tau) - 25I^3\cos(6\omega\tau)] \tag{22}$$

$$P_{51} = \frac{3}{256}[8 + 8I - 6I^2 + I(4 + I)(-4 + 3I)\cos(2\omega\tau) - 10I^2\cos(4\omega\tau) + 5I^3\cos(6\omega\tau)] \tag{23}$$

$$P_{60} = \frac{1}{512}[2(2 + 3I)^2 - 3I(1 + 4I)^2\cos(2\omega\tau) + 30I^2\cos(4\omega\tau) - 5I^3\cos(6\omega\tau)] \tag{24}$$

In the above equations, we set $I \equiv I(\tau)$ for simplicity. Note that once we set $I = 1$, these equations will be degraded to results calculated using the single-mode theory.

[1] M. C. Tichy, H.-T. Lim, Y.-S. Ra, F. Mintert, Y.-H. Kim, and A. Buchleitner, Phys. Rev. A **83**, 062111 (2011).
[2] Y.-S. Ra, M. C. Tichy, H.-T. Lim, O. Kwon, F. Mintert, A. Buchleitner, and Y.-H. Kim, Nat Commun **4**, 2451 (2013).
[3] Y.-S. Ra, M. C. Tichy, H.-T. Lim, O. Kwon, F. Mintert, A. Buchleitner, and Y.-H. Kim, PNAS **110**, 1227 (2013).
